# *Spatholobus suberectus* Exhibits Antidiabetic Activity In Vitro and In Vivo through Activation of AKT-AMPK Pathway

**DOI:** 10.1155/2017/6091923

**Published:** 2017-05-18

**Authors:** Peijun Zhao, Md Badrul Alam, Seok-hyun Lee, Young-Jun Kim, Seul Lee, Hongyan An, Hee-Jeong Choi, Hyeong-U Son, Chul-Hong Park, Hyo-Hyun Kim, Sang-Han Lee

**Affiliations:** ^1^Department of Food Science and Biotechnology, Graduate School, Kyungpook National University, Daegu 41566, Republic of Korea; ^2^Food and Bio-Industry Research Institute, Kyungpook National University, Daegu 41566, Republic of Korea; ^3^Department of Biomedical Sciences, University of North Dakota, Grand Forks, ND 58202-9037, USA; ^4^MR Innovation Co., Ltd., Technopark, Kyungpook National University, Daegu 41566, Republic of Korea

## Abstract

Glucose deposition in peripheral tissue is an important parameter for the treatment of type 2 diabetes mellitus. The aim of this study was to investigate the effects of* Spatholobus suberectus* (Ss) on glucose disposal in skeletal muscle cells and additionally explore its in vivo antidiabetic potential. Treatment of ethanolic extract of* S. suberectus* (EeSs) significantly enhanced the glucose uptake, mediated through the enhanced expression of GLUT4 in skeletal muscle via the stimulation of AKT and AMPK pathways in C2C12 cells. Moreover, EeSs have potential inhibitory action on *α*-glucosidase activity and significantly lowered the postprandial blood glucose levels in STZ-induced diabetic mice, associated with increased expression of GLUT4 and AKT and/or AMPK-mediated signaling cascade in skeletal muscle. Furthermore, administration of EeSs significantly boosted up the antioxidant enzyme expression and also mitigated the gluconeogenesis enzyme such as PEPCK and G-6-Pase enzyme expression in liver tissue of STZ-induced diabetic mice model. Collectively, these findings suggest that EeSs have a high potentiality to mitigate diabetic symptoms through stimulating glucose uptake in peripheral tissue via the activation of AKT and AMPK signaling cascade and augmenting antioxidant potentiality as well as blocking the gluconeogenesis process in diabetic mice.

## 1. Introduction

Diabetes mellitus (DM), a complex and progressive metabolic disorder, may lead to severe complications such as retinopathy, neuropathy, and nephropathy, which severely affect human health and quality of life [1]. According to the International Diabetes Federation (IDF), recent estimates project that DM affects more than 378 million people worldwide and this value is most likely to double by 2035 [2]. Type 1 DM is mainly caused by defective insulin (INS) secretion due to the destruction of pancreatic *β* cells and loss of adipose tissue, whereas type 2 DM is characterized by hyperglycemia, which results from the development of resistance to insulin action, with disturbed carbohydrate/fat metabolism [3]. According to many physicians, adopting healthier lifestyle practices, such as proper exercise and nutrition, can prevent type 2 DM. In addition, therapy involving administration of INS and various oral hypoglycemic drugs, such as sulfonylureas, biguanide, thiazolidinediones, and dipeptidyl peptidase-4 (DPP-4) inhibitor can control DM [4]. However, due to the unwanted side effects, such as hypoglycemia, weight gain, hypersensitivity, gastrointestinal discomfort, nausea, liver and heart failure, and diarrhea, associated with the long-term use of these drugs, the treatment for type 2 DM has been challenging [5]. Therefore, there is a considerable demand for therapeutic agents with high efficacy and few side effects. Thus, research has been extended to identify a proper diet and plant-derived compounds with insulin-like activity that can promote glucose transport and metabolism [6].

Two distinct pathways, phosphatidylinositol-3 kinase (PI3K) and 5′-AMP-activated protein kinase (AMPK), play a pivotal role in glucose transport in the skeletal muscle. PI3K pathway involves the activation of AKT leading to the activation of various downstream enzyme/proteins that promote the translocation of glucose transporter type 4 (GLUT4) from an intracellular pool to plasma membrane [7]. AMPK, a phylogenetically conserved intracellular energy sensor, also plays a central role in GLUT4 translocation by triggering ATP-generating catabolic pathways including *β*-oxidation and glycolysis [8]. Both the pathways are also involved in the phosphorylation and functioning of mitogen-activated protein kinase (MAPK) family components, which play a pivotal role in the activation of insulin-dependent glucose uptake through GLUT4 translocation [9].

Mounting evidence suggests that hyperglycemia leads to an increase in oxidative stress in various tissues through the overproduction of reactive oxygen species (ROS) and triggers various microvascular and macrovascular complications [10]. Several in vivo studies have revealed that oxidative stress due to hyperglycemia plays a pivotal role in the pathogenesis of late diabetic complications such as nephropathy, retinopathy, and neuropathy [11, 12]. In addition, *β*-cells are low in free radical quenching (antioxidant) enzyme, such as superoxide dismutase (SOD), glutathione peroxidase (GPx), and catalase (CAT) [13], as well as ROS-scavenging protein, such as thioredoxin; they are also sensitive to ROS [14]. Thus, medicinal plants, herbs, food, and their chemical constituents with strong antioxidant activity have received considerable attention for their potential to reduce oxidative stress-dependent cellular damage in patients with diabetes.

*Spatholobus suberectus* (Ss), known as Ji Xue Teng in China and Gye-Hyeol-Deung in Korea, is widely used in China as a food supplement in soup, tea, and wine as well as in traditional Chinese medicine for the treatment of anemia, menstrual abnormalities, and arthritis [15]. Phytochemical studies on this herb have revealed various types of polyphenolic compounds as principal constituents, including flavone, isoflavonoids, flavanones, flavanonols, and chalcone [16]. Quinones, steroids, fatty acids, and procyanidins have also been identified in Ss [17]. The extract of Ss stem shows diverse biological functions including antiplatelet [18], anti-inflammatory [19], antioxidant [20], antirheumatic [21], and anticancer [22] activities. However, to the best of our knowledge, no study has investigated the antidiabetic activity of Ss and the underlying mechanisms.

We hypothesized that Ss is functionally associated with GLUT4 translocation involved in the upregulation of glucose uptake. In the present study, we investigated whether Ss suppresses hyperglycemic condition by regulating GLUT4 translocation through the activation of AKT and/or AMPK signaling pathways in vitro and in vivo and by augmenting antioxidant potential in a diabetic mice model.

## 2. Materials and Methods

### 2.1. Plant Material and Extraction

**Figure 1:**
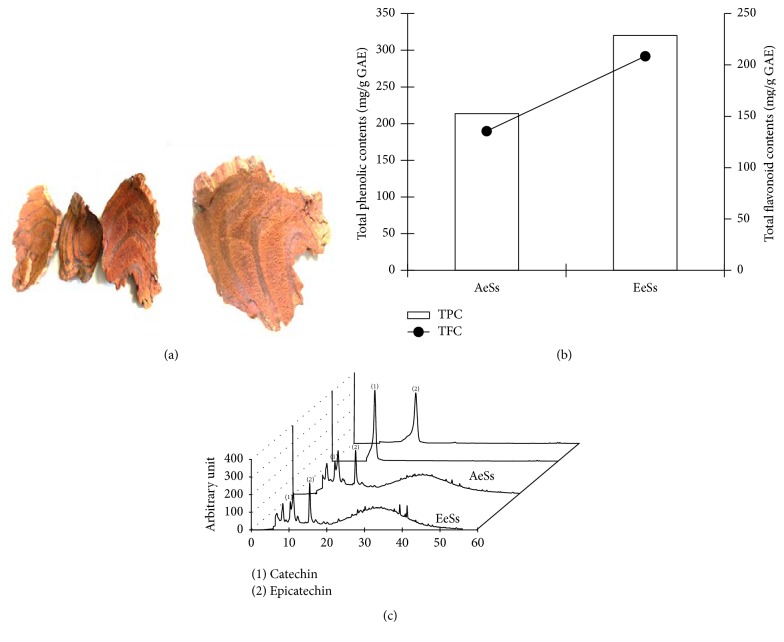
*A classical feature of Spatholobus suberectus and HPLC chromatogram of the extracts monitored at 280 nm*. A classical feature of* Spatholobus suberectus* bark (a) is shown. The total polyphenolic and flavonoid content (b) and the HPLC chromatogram of the major compounds (c) were analyzed as described in detail in Materials and Methods. (1) Catechin and (2) epicatechin. TPC: total polyphenolic contents; TFC: total flavonoid contents; GAE: gallic acid equivalent; AeSs: aqueous extract of* S. suberectus*; EeSs: ethanolic extract of* S. suberectus.*

The stems of Ss were obtained from a Chinese medicinal herb shop in Zhengzhou, China (Figure 1(a)). After air-drying and grinding, 30 g of Ss was subjected to extraction with 15-fold distilled water (1 : 15, w/v) or 10-fold 100% ethanol (1 : 10, w/v) for 12 h in a shaking incubator (E105, Misung Scientific Co., Seoul, Korea) at 60°C and filtered (Filter paper number 1, Whatman Schleicher and Schuell, NH, USA); then, the extracts were lyophilized using a freeze dryer (Ilshin Biobase, Pocheon, Korea) to obtain aqueous extract (AeSs) and ethanolic extract of Ss (EeSs). The powdery extracts were dissolved in DMSO and stored in the dark at 4°C. The plant was taxonomically identified by comparing morphological observation and rRNA sequencing which was conducted by Dr. J. C. Heo, a specialist of plant taxonomy, Keimyung University, Daegu, Korea. The voucher specimen (2016-Ss) is deposited in our laboratory for future reference.

### 2.2. Drugs and Chemicals

2,2-Diphenyl-1-picrylhydrazyl (DPPH), neocuproine, 2,4,6-tris(2-pyridyl)-s-triazine, 2,2′-azino-bis(3-ethylbenzthiazoline-6-sulfonic acid) (ABTS), *α*-glucosidase, p-nitrophenyl-*α*-D-glucopyranoside (pNPG), 3-(4,5-dimethylthiazol-2-yl)-2,5-diphenyltetrazolium bromide (MTT), dimethyl sulfoxide (DMSO), and 6-hydroxy-2,5,7,8-tetramethylchroman-2-carboxylic acid (Trolox) were purchased from Sigma-Aldrich (St. Louis, MO, USA). Dulbecco's modified Eagle's medium (DMEM), fetal bovine serum (FBS), penicillin-streptomycin mixture, and 0.25% trypsin-ethylenediaminetetraacetic acid (EDTA) were purchased from GE Healthcare Life Sciences (Hyclone, Mordialloc, VIC, Australia). 2-NBDG was purchased from Thermo Fisher Scientific Inc. (Carlsbad, CA, USA). Antibodies were purchased from Bioworld Technology, Inc. (St. Louis Park, MN, USA), including anti-protein kinase B (PKB/AKT), anti-AMP-activated protein kinase (AMPK), anti-glucose transporter type 4 (GLUT4), and anti-*β*-actin.

### 2.3. High-Performance Liquid Chromatography with Diode-Array Detection Analysis

Phytochemical characteristics of AeSs and EeSs were studied via high-performance liquid chromatography (HPLC) by using standard compounds, including catechin and epicatechin. HPLC was performed using an Agilent 1200 chromatographic system (Agilent Technologies, Santa Clara, CA, USA) with a UV-Vis diode-array detector and the ChemStation software (version G2170BA, Agilent Technologies). The samples were filtered through a 0.45 *μ*m nylon filter (E0034, Análisis Vínicos, Tomelloso, Spain), and polyphenolic compounds were analyzed according to the method described by Kumar et al. [23]. A Zorbax C18 column (250 × 4.6 mm, 5-*μ*m particle size) (Agilent Technologies) was maintained at 30°C, and elution was performed with a linear gradient of solvent A (water) and solvent B (0.02% trifluoroacetic acid in acetonitrile) as follows: 0–20 min, 80% of A; 20–35 min, 40% of A; 35–50 min, 10% of A; 50–70 min, 0% of A; and 70–85 min, 20% of A at *λ* = 280 nm. The flow rate was 0.8 mL/min, and the injection volume was 10 *μ*L. Polyphenolic compounds were identified via comparison of retention times with those of available pure standards.

### 2.4. In Vitro Antioxidant Activity Assays

#### 2.4.1. 2,2-Diphenyl-1-picrylhydrazyl Radical-Scavenging Assay

The DPPH radical-scavenging assay used for the evaluation of free radical-scavenging activity of the AeSs and EeSs was conducted following the protocol described elsewhere [24]. Briefly, 198 *μ*L of a 0.2 mM solution of DPPH in 50% ethanol was added to 2 *μ*L of various concentrations (10, 30, and 100 *μ*g/mL) of the samples. The mixture was allowed to stand at 25°C for 10 min in dark place and the absorbance was measured at 517 nm in a microplate reader (Victor3, PerkinElmer, Turku, Finland). Ascorbic acid was checked as a standard compound. The percent inhibition activity was calculated using the following equation:

(1) Radical-scavenging  activity  %  of  inhibition=Abscontrol−AbssampleAbscontrol×100,

where Abs_control_ is the absorbance of the control and Abs_sample_ is the absorbance of the sample/standard. All samples were analyzed in triplicate.

#### 2.4.2. 2,2′-Azino-bis(3-ethylbenzthiazoline-6-sulfonic acid) Radical-Scavenging Assay

The method described by Re et al. [25] was adopted for the ABTS radical-scavenging assay. Various concentrations (10, 30, and 100 *μ*g/mL) of the sample were allowed to react with 198 *μ*L of the ABTS^•+^ solution, and the absorbance was measured at 734 nm. Ascorbic acid was tested as a positive antioxidant compound. The percent inhibition activity was calculated using (1).

#### 2.4.3. Ferric Reducing Antioxidant Power Assay

For measuring the reducing power, the ferric reducing antioxidant power (FRAP) assay was performed as previously described [26], with a slight modification. Two microliters of the aqueous sample at varying concentrations (10, 30, and 100 *μ*g/mL) and 198 *μ*L of FRAP reagent were mixed, and the absorbance was recorded at a 595 nm. Ascorbic acid was tested as a standard antioxidant compound, and the ascorbic acid equivalent FRAP value (*µ*M) was calculated from a standard curve generated for ascorbic acid.

#### 2.4.4. Cupric Reducing Antioxidant Capacity

The CUPRAC of LCBE was determined according to the method described by Apak et al. [27], with slight modifications. A solution of 10 mM CuCl2, 7.5 mM neocuproine, and 1 M ammonium acetate buffer (pH 7.0) was added and the resultant solution was mixed to the indicated concentration (10, 30, and 100 *μ*g/mL) of the samples. Following a 1-h incubation period at 25°C, the absorbance was measured at 450 nm. The ascorbic acid equivalent CUPRAC value (*µ*M) was calculated from a standard curve generated for ascorbic acid.

### 2.5. *α*-Glucosidase Inhibitory Assay

The *α*-glucosidase inhibition activity of AeSs and EeSs was measured according to the method described by Kim et al. [28]. In brief, 2 *μ*L of predetermined concentration of AeSs and EeSs were mixed with 0.2 U/mL *α*-glucosidase in 0.1 M sodium phosphate buffer (pH 7.0); the mixture was incubated at 37°C for 10 min. Subsequently, pNPG (2 mM in the above buffer, pH 7.0), acting as a substrate, was added to the solution to initiate the enzyme-substrate reactions at 37°C for 1 h. The enzyme-substrate reaction was performed at 37°C for 30 min. The *α*-glucosidase inhibition activity was measured spectrophotometrically at 405 nm on a microplate reader (Perkin Elmer, Wallac Victor3, MA, USA) using a 96-well plate. Acarbose served as the positive control and percent inhibition was calculated using (1).

### 2.6. Cell Culture and Cell Viability Assay

C2C12 cells (American Type Culture Collection, Manassas, VA, USA) were cultured at 37°C in DMEM supplemented with 10% FBS and streptomycin-penicillin (100 *µ*g/mL and 100 U/mL, resp.; Hyclone, Mordialloc, VIC, Australia) in a humidified atmosphere of 5% CO_2_. The tetrazolium dye colorimetric test (MTT) was used to determine the viability of C2C12 cells. C2C12 cells were first cultured in 96-well plates (1 × 10^5^ cells/well) for 24 h. After reaching 90% confluence, they were treated with various concentrations of AeSs and EeSs. After 24 h of incubation, the MTT reagent was added to each well, and the plate was incubated at 37°C for 1 h. The media were removed, and the wells were washed twice with PBS (pH 7.4). The intracellular insoluble formazan was dissolved in 100% DMSO. The absorbance in each well was measured at 570 nm using a microplate reader (Perkin Elmer, Wallac Victor3, MA, USA), and the percentage of viability was calculated.

### 2.7. Muscle Differentiation and Glucose Uptake Assay

C2C12 cells were cultured in 96-well plates (1 × 10^5^ cells/well) with DMEM containing 10% FBS and 1% P/S at 37°C and exposed to 5% CO_2_ atmosphere. When the cells reached confluence, they were subjected to differentiation in DMEM supplemented with 2% horse serum for 5 days. Then, the cells were starved in low-glucose serum-free DMEM for 24 h. Subsequently, the 2-NBDG assay was performed to evaluate glucose uptake [22]. In brief, cells were treated with various concentrations of AeSs and EeSs and insulin (100 nM) for 12 h, followed by 20 *µ*M of 2-NBDG for 24 h. After incubation, the cells were washed thrice with cold PBS and 2-NBDG uptake was measured with a fluorometer (Perkin Elmer, Wallac Victor3, MA, USA) at excitation and emission wavelengths of 490 and 535 nm, respectively.

### 2.8. Animal Experiments and STZ-Induced Diabetes

Six-week-old male C57BL/6J mice (Samtaco, Osan, Korea) were used in this experiment and maintained in a temperature- and humidity-controlled room (22 ± 1°C and 55 ± 5%, respectively) with a 12-h light/dark cycle. They had free access to food and water ad libitum. All animals (20 mice) were quarantined at the Animal Care Center, KNU, and acclimatized to the laboratory environment for 1 week before the experiment. As per the Guidelines of the Committee on Laboratory Animal Ethics (Chair: Hee-Kyung Jin, College of Veterinary Medicine, Kyungpook National University), we strictly kept all guidelines recommended by the Committee which was approved as KNU Approval number 2016-122 in accordance with Committee for the Update of the Guide for the Care and Use of Laboratory Animals, Institute for the Laboratory Animal Research, Washington, DC, USA. Animals were randomly divided into four groups, each containing five animals: Normal Control (NC), streptozotocin- (STZ-) induced diabetic control (SDC), STZ-induced diabetic plus acarbose (200 mg/kg bw) (SDA), and STZ-induced diabetic plus EeSs (100 mg/kg bw) (SDEeSs). Diabetes was induced in the diabetic group by intraperitoneal injection of STZ dissolved in 50 mM citrate buffer (pH 4.5) at 50 mg/kg for five consecutive days. The NC group was injected with an equal amount of citrate buffer. After 5 days, fasting blood glucose (FBG) levels were measured and mice with levels ≥200 mg/dL (11.1 mmol/L) were selected for this study.

### 2.9. Oral Glucose Tolerance Test (OGTT)

To examine the effects of EeSs on glucose tolerance, an oral glucose tolerance test (OGTT) was performed after overnight fasting. To perform this test, a single dose of glucose solution (1 g/kg) and EeSs (200 mg/kg) were orally administered to each mice and the subsequent levels of blood glucose were measured by glucometer (Roche Diagnostics, ACCU-CHEK® Active, Mannheim, Germany) at 0, 0.5, 1, 1.5, 2, 2.5, and 3 h after the administration of glucose solution.

### 2.10. Reverse Transcription-Polymerase Chain Reaction (RT-PCR)

Total RNA was extracted using TRIzol (Invitrogen, Carlsbad, CA, USA) according to the manufacturer's instructions. Total RNA (2 *µ*g) was reverse transcribed using reverse transcriptase (MP Biomedicals, Santa Ana, CA, USA) and oligo(dT) primers. cDNA was amplified using a thermal cycler Dice TP600 (Takara Bio, Inc., Otsu, Shiga, Japan). Polymerase chain reaction (PCR) products were visualized via ethidium bromide staining after electrophoresis. Specific oligonucleotide primers for mouse transcripts were used (forward: GAGATGGTCCACCTGAAGGA; reverse: ATCAGGTTCCGAACAGTTGC for* Insr*; forward: AAGCACCTGGTGGCTCTCTA; reverse: TCAGGATAACCTGCCAGACC for* Irs-1*; forward: ACGCATATACCCGCTACCTG; reverse: TCCTCTGTCAGCATCACCTG for* Hmox-1*; forward: CAGTGAAGACCAACCCCAAT; reverse: CCTTCAATGGGAACACCTTC for* Pepck*; forward: CTGGCCCATTCAGAGAAGAC; reverse: GTCTGCAGCTTCCAGCTTCT for* Nqo-1*; forward: ACACCGAGATGAACGATCTG; reverse: ATGTACTTGGGGTCGGTCAT for* Gpx-1*; forward: AAGCGGTGAACCAGTTGTGT; reverse: GCCAATGATGGAATGCTCTC for* Sod1*; forward: GCCAACAAGATTGCCTTCTC;* reverse: *AGGAATCCGCTCTCTGTCAA for* Cat*; forward: GTCCAATGTCCTTGCTCCAG; reverse: CGTCGTCCAGCTCGTTCTAC for* Glut4*; forward: TTTGGGATCCAGTCAACACA, reverse: GCACGGAAGTGTTGCTGTAG for* G6Pase*).

### 2.11. Preparation of Protein Lysates and Western Blot Analysis

After the experiments were completed, the C2C12 myotubes, mice skeletal muscle, and liver tissue lysates were prepared using a standard protocol. In brief, the cell and tissue samples were mixed with sample buffer (250 mM tris-hydrochloride (pH 6.8), 0.5 M dithiothreitol (DTT), 10% sodium dodecyl sulfate (SDS), 0.5% bromophenol blue, 50% glycerol, and 5% 2-mercaptoethanol) and denatured at 95°C for 5 min. Sample proteins (50 *µ*g) were separated via 10% SDS-polyacrylamide gel electrophoresis (PAGE) and electrotransferred to nitrocellulose transfer membranes (Whatman, 401396, Dassel, Germany), which were incubated overnight at 4°C with 5% skim milk or 5% bovine serum albumin and a range of antibodies. Anti-AMPK, anti-phospho-(p)-AMPK, anti-AKT, anti-p-AKT, anti-GLUT4 (Bioworld Technology), and anti-*β*-actin (Bethyl Laboratories) antibodies were the primary antibodies used, and a horseradish peroxidase- (HRP-) conjugated goat anti-rabbit immunoglobulin G (IgG) (Santa Cruz Biotechnology) was used as the secondary antibody. The antigen-antibody reaction was detected using a SuperSignal West Femto maximum sensitivity substrate (Thermo Fisher Scientific, Rockford, IL, USA).

### 2.12. Statistical Analysis

All in vitro data are expressed as the mean ± standard deviation (SD) and were analyzed using paired Student's* t*-test. Differences were considered significant at *p* < 0.05 and *p* < 0.01. All in vivo data are expressed as the mean ± standard error of the mean (SEM) and were analyzed using a one-way analysis of variance (ANOVA), followed by the least significant differences (LSD) test. Differences were considered significant at *p* < 0.05, *p* < 0.01, and *p* < 0.001. All analyses were performed using the statistical package for the social sciences (SPSS) version 23.0 (SPSS, Chicago, IL, USA).

## 3. Results

### 3.1. HPLC Analysis of* Spatholobus suberectus* (Ss) Extract

To identify the active components of the aqueous and ethanolic extracts of Ss (AeSs and EeSs, resp.), total phenolic contents and total flavonoid content of each extract were carried out. The results showed that total phenolic contents and total flavonoid contents of AeSs were 68% and 64% of those of EeSs, respectively (Figure 1(b)). HPLC analysis was performed using catechin and epicatechin as standards. Interestingly, AeSs and EeSs showed peaks with the same retention time as catechin (10.12 min) and epicatechin (13.25 min), respectively (Figure 1(c)).

### 3.2. Radical-Scavenging Activities of Ss Extract

To investigate the antioxidant properties of Ss, its potential to scavenge DPPH^•^ and ABTS^•+^ was measured. Both AeSs and EeSs significantly scavenged DPPH^•^ and ABTS^•+^ in a dose-dependent manner (Table 1). In addition, to investigate whether Ss had electron-donating potential, cupric reducing antioxidant capacity (CUPRAC) and FRAP assays were performed. Both AeSs and EeSs showed a strong and concentration-dependent reducing power (Table 1). Based on these observations, we speculated that both AeSs and EeSs have a strong capacity to scavenge various free radicals through hydrogen atom transfer and/or electron donation.

### 3.3. *α*-Glucosidase Inhibitory Activity of Ss Extracts

**Figure 2:**
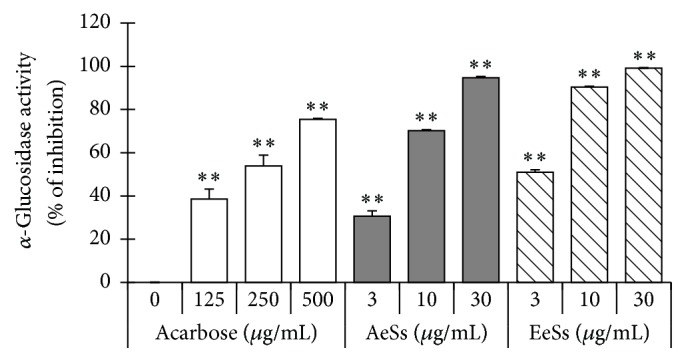
*α*-*Glucosidase inhibitory activity of Spatholobus suberectus*. Results are mean ± standard deviation (SD) of triplicate experiments. ^*∗∗*^*p* < 0.01 versus control using Student's *t*-test. AeSs: aqueous extract of* S. suberectus*; EeSs: ethanolic extract of* S. suberectus*.

*α*-Glucosidase, the key digestion enzyme that converts carbohydrates to glucose in the intestine, acts as a therapeutic target for type 2 DM by reducing postprandial hyperglycemia [3]. In this study, both Ss extracts (AeSs and EeSs) significantly inhibited *α*-glucosidase activity in a concentration-dependent manner (IC_50_ = 6.42 ± 1.45 *μ*g/mL and 2.81 ± 0.48 *μ*g/mL, resp.) while acarbose, a well-known *α*-glucosidase inhibitor, showed an IC_50_ value of 217.87 ± 0.21 *μ*g/mL (Figure 2).

### 3.4. Ss Extract Enhances Glucose Uptake and GLUT4 Activation via PI3K/AKT and AMPK Pathway

**Figure 3:**
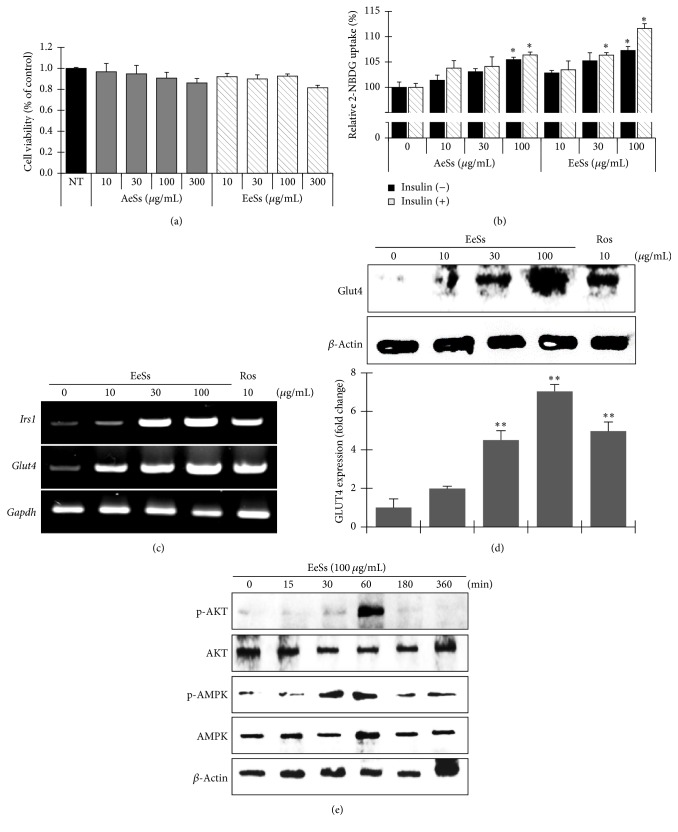
*Cell viability and glucose uptake associated with signaling activity of Spatholobus suberectus in C2C12 cells. *C2C12 cells were seeded at a density of 2 × 10^5^ cells per well (96-well plate), and then the MTT assay (a) was performed. Differentiated C2C12 cells were incubated with AeSs and EeSs with or without insulin at indicated concentration for 24 hr and the glucose uptake (b) was measured as described in Materials and Methods. (c) Differentiated C2C12 cells were collected and mRNA levels of genes were determined by RT-PCR; (d and e) protein extracts were prepared and subjected to western blot assay using indicated primary antibody; beta-actin levels were used as a control for equal loading (d and e). ^*∗∗*^*p* < 0.01 versus control; ^*∗*^*p* < 0.05 versus control using Student's* t*-test. NT: not treated; Ros: rosiglitazone-treated; AeSs: aqueous extract of* S. suberectus*; EeSs: ethanolic extract of* S. suberectus.*

The glucose uptake and viability of the C2C12 myotubes were examined following exposure to AeSs and EeSs. Treatment with AeSs and EeSs at concentrations of 10–300 *µ*g/mL had no cytotoxic effect on C2C12 myotubes (Figure 3(a)). Next, to establish the regulatory role of AeSs and EeSs in glucose metabolism or consumption of muscle cells, glucose uptake assay was performed using 2-(N-(7-nitrobenz-2-oxa-1,3-diazol-4-yl)amino)-2-deoxyglucose (2-NBDG) in C2C12 myotubes. Interestingly, glucose uptake was considerably stimulated by the treatment of both AeSs and EeSs with or without insulin in a concentration-dependent manner (Figure 3(b)). These results suggest that the Ss extract is involved in glucose uptake signaling pathways in muscle cells. In skeletal muscle cells, GLUT4 plays a pivotal role in the uptake of glucose under insulin-dependent and/or basal states [29]. To further elucidate the possible molecular mechanism of action of EeSs, the transcription level of* Irs-1* and* Glut4* expression was examined. Interestingly, treatment with EeSs significantly amplified* Irs-1* as well as* Glut4* mRNA expression in a concentration-dependent manner (Figure 3(c)). The protein level of GLUT4 was determined by western blot analysis (Figure 3(d); quantifications and statistical analyses are presented in adjacent graphs).

The insulin signaling pathway, which involves the activation of PI3K/AKT and insulin-independent AMPK pathway, may have the central role of glucose transport in the skeletal muscles through GLUT4 translocation [9]. Thus, to evaluate the capacity of EeSs to activate AKT or AMPK signaling pathway, C2C12 myotubes were incubated with optimized concentration of EeSs and the time-dependent phosphorylation of AKT and/or AMPK was analyzed. Treatment with EeSs caused a significant increase in both AKT and AMPK phosphorylation at 60 min in C2C12 cells, which recovered to normal levels on prolonged exposure (Figure 3(e)). From these findings, we concluded that EeSs by itself shows the insulin mimetic effect as well as activating the insulin-independent pathway to induce glucose uptake in the skeletal muscles.

### 3.5. In Vivo Antidiabetic Activity of EeSs in Streptozotocin- (STZ-) Induced Diabetic Mice

**Figure 4:**
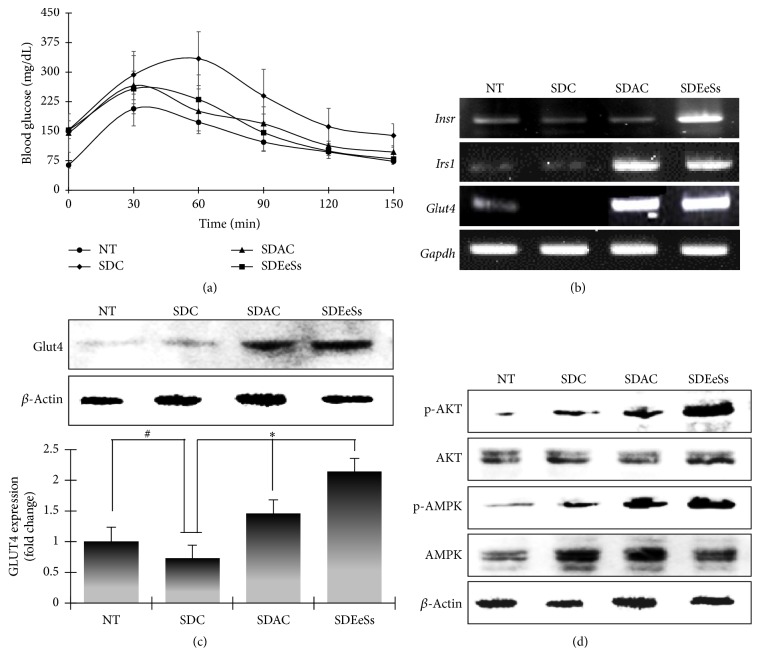
In vivo* antidiabetic activity of Spatholobus suberectus*. STZ-induced diabetic mice were treated with EeSs or acarbose at 200 mg/kg dose and oral glucose tolerance test was monitored at various time intervals (a) after an oral load of glucose (2 gm/kg). After treatment, the skeletal muscle tissues were excised and RT-PCR analysis (b) was performed, followed by western blot analysis (c and d). NT: not treated; SDC: STZ-induced diabetic control mice; SDAC: STZ-induced diabetic acarbose-treated mice; SDEeSs: STZ-induced diabetic EeSs-treated mice. ^#^*p* < 0.05 versus NT; ^*∗*^*p* < 0.05 versus SDC group using ANOVA followed by LSD test.

STZ, the most widely used diabetogenic agent in animal models, is used for selective destruction of pancreatic *β*-cells or impairment of sensitivity of insulin for glucose uptake [29]. EeSs significantly increased glucose uptake in C2C12 cells. We examined its role in controlling hyperglycemia in STZ-induced diabetic mice. In the EeSs-treated group (SDEeSs), a significant decline in blood glucose level was observed after glucose load (1 g/kg) (Figure 4(a)). These results revealed that EeSs has the potential to decrease blood glucose levels (by 43%) at 150 min in the STZ-induced diabetic mice at a dose of 200 mg/kg body weight. In the acarbose-treated group (SDAC), which is a well-known *α*-glucosidase inhibitor, blood glucose level decreased by 30% after 150 min of treatment at the same dose.

### 3.6. EeSs Acts on Insulin Mimetic Effects in Streptozotocin- (STZ-) Induced Diabetic Mice

To assess the insulin mimetic effect of EeSs in vivo,* Insr*,* Irs-1*, and* Glut4* mRNA expression was determined using RT-PCR. As expected, in the SDEeSs group, there was a significant increase in the transcription levels of* Insr*,* Irs-1*, and* Glut4 *(Figure 4(b)). Western blot analysis of GLUT4 also revealed the expected expression (Figure 4(c); quantifications and statistical analyses are presented in adjacent graphs). To understand the underlying mechanism, AKT and AMPK phosphorylation in the skeletal muscles of STZ-induced diabetic mice was examined. The analysis revealed that SDEeSs caused a significant increase in AKT and AMPK phosphorylation in the skeletal muscles (Figure 4(d)), which validated our in vitro findings.

### 3.7. EeSs Treatment Mitigates the Oxidative Stress in Streptozotocin- (STZ-) Induced Diabetic Mice

Several in vivo studies suggested that oxidative stress is associated not only with diabetic complications but also with insulin resistance and that antioxidant therapy improves insulin sensitivity in a diabetic animal model [30]. Thus, to examine whether EeSs has the capacity to mitigate oxidative stress in an STZ-induced diabetic animal model, mRNA expression of endogenous primary antioxidant enzymes, such as* Sod1*,* Gpx-1*, and* Cat*, as well as phase II antioxidant enzymes, such as heme oxygenase 1* (Hmox-1)* and NAD(P)H:quinone oxidoreductase* (Nqo1)*, was examined using RT-PCR. Interestingly, in the STZ-induced diabetic control (SDC) group, the antioxidant enzyme expression was completely mitigated, whereas in the SDEeSs treatment group, the expression of both primary and phase II antioxidant enzyme increased in the liver of STZ-induced diabetic mice. This suggested that the antioxidant capacity of EeSs may partially contribute to the improvement of glucose uptake by the skeletal muscles as well as reduce blood glucose levels in the STZ-induced diabetic model.

### 3.8. EeSs Treatment Abolished the Gluconeogenesis in Streptozotocin- (STZ-) Induced Diabetic Mice

Impaired insulin secretion and peripheral insulin resistance cause hyperglycemia with increased hepatic glucose production in patients with type 2 DM [26]. Phosphoenolpyruvate carboxykinase (PEPCK) and glucose-6-phosphatase (G-6-Pase), the key gluconeogenic enzyme, play a crucial role in de novo* synthesis* of glucose (gluconeogenesis) in the liver from precursors, such as lactate, pyruvate, and glycerol, which maintain blood glucose concentration during starvation and diabetic condition [27]. Thus, to assess the effect of EeSs on gluconeogenesis in STZ-induced diabetic mice, the mRNA expression of* Pepck* and* G-6-Pase* in the liver was examined using RT-PCR. In the SDC group, there was an increase in* Pepck *and* G-6-Pase* mRNA expression, whereas in the SDEeSs group, the expression decreased. These findings suggested that EeSs alleviated the hyperglycemic condition, partly by abolishing gluconeogenesis in STZ-induced diabetic mice.

## 4. Discussion

Since ancient times, plants have been used in the treatment of various diseases. Plants are believed to have less toxic effects and considered an ideal source for drug development [31]. However, there is scarce scientific evidence supporting the efficacy of plant derivatives, and in most cases, their mode of action is poorly understood. In this study, the antidiabetic potential of Ss was evaluated using in vitro and in vivo models. To the best of our knowledge, this study demonstrated for the first time the underlying mechanism by which Ss alleviated the hyperglycemic condition by activating GLUT4 through the activation of AKT and AMPK signaling in C2C12 cells as well as in a STZ-induced diabetic mice model. In addition, treatment with Ss augmented the antioxidant capacity and inhibited gluconeogenesis in STZ-induced diabetic mice.

The most remarkable feature of type 2 DM is insulin resistance. Thus, development of novel insulin-sensitizing agents is gaining momentum [32]. Skeletal muscle is a critical factor in insulin-dependent glucose uptake, disposal, and storage. Moreover, the augmentation of glucose transport to this tissue is believed to be a central role in the regulation of glucose homeostasis and management of type 2 DM [33]. In this study, the glucose uptake potential of Ss was explored using C2C12 cells. The exposure to Ss enhanced glucose uptake in C2C12 cells in a concentration-dependent manner. On exposure to insulin, synergistic effects of Ss on glucose uptake were observed, confirming that Ss have the capacity to mimic the insulin-like activity (Figure 3(b)). In the skeletal muscle cells, GLUT4, the major glucose transporter, plays a central role in glucose uptake in insulin-dependent and insulin-nondependent state [34]. The treatment of EeSs significantly enhanced the cellular expression of GLUT4 at the transcriptional and translation levels, which suggests that glucose uptake by EeSs is involved in the increase of GLUT4 expression in C2C12 cells and the muscle tissues of STZ-induced diabetic mice (Figures 3(c), 3(d), 4(b), and 4(c)).

The continuous transportation of glucose molecules by GLUT4 is mainly regulated by two distinct mechanisms: (1) insulin-dependent signaling pathways involving PI3K/AKT activation and (2) insulin-independent pathways involving AMPK activation [33]. Therefore, to unravel the molecular mechanism by which EeSs augments glucose utilization in C2C12 cells and STZ-induced diabetic mice, its effect on insulin-dependent glucose utilization was investigated. In skeletal muscles, the binding of insulin to its receptor phosphorylates tyrosine residues on intracellular substrates including the insulin receptor substrate 1 (IRS1) and consequently activates a complex network of signaling molecules, such as phosphatidylinositol-3-kinase (PI-3-kinase), and its downstream target AKT [7], which is important for the progression of downstream signaling cascade that leads to physiological responses, such as glucose uptake and glucogen storage [35]. In this study, EeSs treatment activated the AKT phosphorylation at 60 min in C2C12 cells and in STZ-induced diabetic mice (Figures 3(e) and 4(d), resp.). In addition, EeSs treatment increased the transcription levels of* Insr* and* Irs-1* (Figures 3(c) and 4(b)). These results collectively suggested that EeSs may show insulin mimetic activity to stimulate glucose utilization in skeletal muscle cells in vitro and in vivo. Furthermore, AMPK is an intracellular energy sensor and regulates the whole body's energy balance; it has been investigated as a potential therapeutic target for type 2 DM. The activation of AMPK leads to GLUT4 translocation and eventually glucose utilization, independent of insulin action [36]. In this study, the exposure of C2C12 cells to EeSs resulted in the phosphorylation of AMPK at 60 min compared with the untreated group. In vivo, AMPK in the mice skeletal muscles was examined via western blot analysis; the results were consistent with in vitro findings. These data prove that EeSs upregulates p-AMPK levels, promoting the expression of GLUT4 and thereby lowering blood glucose levels in mice. Natural compounds with insulin mimetic activity are being considered as potential therapeutic agents for the treatment/or management of type 2 DM, such as berberine enhanced insulin-mediated GLUT4 translocation and glucose transport through the activation of AKT and AMPK pathways [37]. Catechin and epicatechin, the principle constituents of tea, significantly increased the glucose uptake activity in the presence of insulin through AKT activation [38]. HPLC analysis revealed that both catechin and epicatechin were present in EeSs (Figure 1(c)). Therefore, the antidiabetic effect of EeSs is mediated at least in part by the activation of AKT and/or AMPK; this finding is supported by the results of a previous study [39].

**Figure 5:**
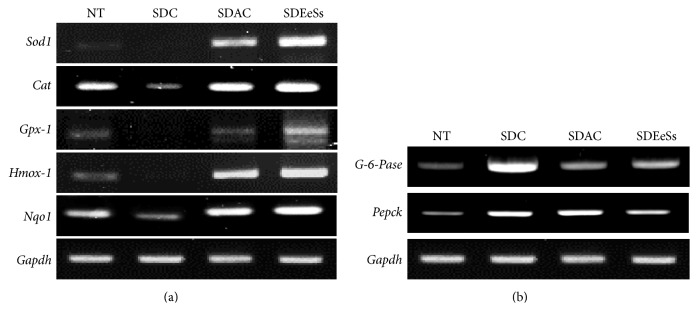
*Effects of Spatholobus suberectus on antioxidant and gluconeogenesis-related enzymes*. STZ-induced diabetic mice were treated with EeSs or acarbose at 200 mg/kg; after treatment, the liver tissue was excised. This was followed by RT-PCR analysis of antioxidant (a) and gluconeogenesis-related enzymes (b). NT: not treated; SDC: STZ-induced diabetic control mice; SDAC: STZ-induced diabetic acarbose-treated mice; SDEeSs: STZ-induced diabetic EeSs-treated mice.

Diabetes is associated with indices of oxidative damage, which can lead to the glycation of tissue proteins and glucose autoxidation and consequently the generation of hydrogen peroxides, hydroxyl radicals, and protein-reactive ketoaldehydes, as well as also increased lipid peroxidation, superoxide production, and oxidative DNA damage [40]. Moreover, the formation of advanced glycation end products (AGEs) activates the transcription factor nuclear factor kappa B (NF-*κ*B) and its various downstream target genes, leading to the overproduction of nitric oxide (NO), which is believed to be mediator of *β*-cell damage [41]. Thus, antioxidants can protect against oxidative stress and have beneficial implications in diabetes management. In the present study, EeSs showed a strong antioxidant potential in in vitro antioxidant model (Table 1). Mounting evidence suggests that phenolic compounds, commonly found in edible and inedible plants, have diverse biological effects, including antidiabetic and antioxidant activity, mainly due to their redox properties, which play an important role in adsorbing and neutralizing free radicals [42]. Flavonoids, one of the most diverse and widespread groups of natural compounds, are likely to be the most important natural phenolics and possess a broad spectrum of chemical and biological activities, including radical-scavenging properties [43]. Thus, it was important to determine the concentration of total phenolic compounds (327.07 ± 1.22 mg gallic acid equivalent per gram of dry weight) and flavonoids (218.06 ± 0.24 mg catechin equivalent per gram of dry weight) in the plant chosen for this study (Figure 1(c)). The correlation between the content of polyphenols and flavonoids and the antioxidant activity and *α*-glucosidase activity was calculated using the Pearson coefficient (*ρ*) and linear regression analysis (data not shown). The results showed very strong correlations with DPPH and ABTS radical-scavenging activities (*ρ* = 0.966 and 0.998, resp.) but moderate ones in *α*-glucosidase activity (*ρ* = 0.636). Furthermore, primary antioxidant enzymes, such as SOD, CAT, and GPx, as well as phase II antioxidant enzymes, such as HO-1 and NQO1, were mitigated in the SDC group than in the NC group, possibly due to the increased glucose oxidation, formation of AGE-mediated free radical generation, and NO donor property of STZ [44]. Oral administration of EeSs boosted the expression of* Sod1*,* Cat*,* Gpx-1*,* Hmox-1*, and* Nqo-1* mRNA levels in the diabetic mice liver (Figure 5(a)), suggesting that EeSs has a strong antioxidant potential to quince free radicals and hence the ability to prevent diabetes-associated complication.

Hepatic gluconeogenesis is essential for maintaining blood glucose levels during fasting and is the major contributor to postprandial and fasting hyperglycemia in diabetes [45]. It is noteworthy that the abandoned expression and activity of gluconeogenic enzymes increase gluconeogenesis in diabetes. Normally, insulin can mitigate gluconeogenesis through multiple mechanisms; one of them is the direct suppression of the transcription of key gluconeogenic genes, such as* PEPCK* and* G-6-Pase*, by blocking the recruitment of transcriptional coactivators* PGC-1α* and CREB-binding proteins to the promoters of the* PEPCK* and* G-6-Pase* genes [46]. In the present study, the administration of EeSs reduced the expression of* PEPCK* and* G-6-Pase* in liver compared with the STZ-induced diabetic control mice group (Figure 5(b)). These findings also revealed that EeSs shows a strong insulin mimetic activity and decreased the blood glucose level not only enhancing the expression of GLUT4 expression but also mitigating the gluconeogenesis process in diabetic mice liver, possibly in part through the activation of AKT and/or AMPK pathway. However, further mechanistic studies are required.

## 5. Conclusion

All these beneficial effects of EeSs are especially hopeful in preventing hyperglycemia. Overall, EeSs had greater antidiabetic effects compared to the corresponding crude aqueous extracts implying that compounds responsible for these activities are polar in nature. EeSs has a better antioxidant potential than AeSs, and this was probably due to the phenolic compounds. In conclusion, this study has undoubtedly provided scientific confirmation and evidence for the safe use of* Spatholobus suberectus* by traditional healers on the preventive/curative purposes for diabetic symptoms. However the nature of the active principle(s) responsible for all these positive effects requires further investigation.

## Figures and Tables

**Table 1:** Antioxidant activity of AeSs and EeSs in various in vitro assay systems.

Compound	Concentration	DPPH^(1)^	ABTS^(2)^	FRAP^(3)^	CUPRAC^(4)^
		Activity (% of control)			
AA (*μ*M)	25	36.3 ± 0.026^*∗∗*^	10.5 ± 0.009^*∗∗*^	405.5 ± 0.011^*∗∗*^	456.9 ± 0.003^*∗∗*^
	50	55.3 ± 0.093^*∗*^	29.3 ± 0.009^*∗∗*^	706.9 ± 0.011^*∗∗*^	788.2 ± 0.010^*∗∗*^

AeSs (*μ*g/mL)	10	21.9 ± 0.020^*∗∗*^	6.8 ± 0.004^*∗*^	229.2 ± 0.007^*∗∗*^	457.8 ± 0.025^*∗∗*^
	30	28.1 ± 0.018^*∗∗*^	6.9 ± 0.002^*∗∗*^	436.3 ± 0.027^*∗∗*^	829.2 ± 0.005^*∗∗*^
	100	48.5 ± 0.043^*∗∗*^	39.5 ± 0.012^*∗∗*^	1207.1 ± 0.101^*∗∗*^	1330.0 ± 0.005^*∗∗*^

EeSs (*μ*g/mL)	10	16.3 ± 0.003^*∗*^	4.0 ± 0.001^*∗∗*^	271.0 ± 60.004^*∗∗*^	490.7 ± 0.014^*∗∗*^
	30	22.9 ± 0.012^*∗*^	11.4 ± 0.002^*∗∗*^	608.8 ± 0.011^*∗∗*^	914.7 ± 0.019^*∗∗*^
	100	35.4 ± 0.020^*∗∗*^	46.6 ± 0.030^*∗∗*^	1211.5 ± 0.076^*∗∗*^	1373.5 ± 0.011^*∗∗*^

^(1)^DPPH-radical scavenging assay; ^(2)^ABTS-radical scavenging activity assay; ^(3)^ferric reducing antioxidant power assay; ^(4)^cupric(II) ion reducing antioxidant capacity assay. Values are expressed as mean ± SD of triplicate determinations; ^*∗*^*p* < 0.05 and ^*∗∗*^*p* < 0.01 versus control using the Student *t*-test. AA: ascorbic acid; AeSs: aqueous extract of *S. suberectus*; EeSs: ethanolic extract of *S. suberectus.*

## Additional Points

*Statement of Human Rights*. This article does not contain any studies with human participants performed by any of the authors.
